# Spontaneous Spinal Subdural Hematoma Mimicking Myocardial Infarction

**DOI:** 10.7759/cureus.9486

**Published:** 2020-07-30

**Authors:** Hammad Ghanchi, Imran Siddiqi, Ariel Takayanagi, Tye Patchana, Faris J Fakhoury

**Affiliations:** 1 Neurosurgery, Riverside University Health System Medical Center, Moreno Valley, USA; 2 Neurosurgery, College of Osteopathic Medicine, Western University of Health Sciences, Pomona, USA; 3 Neurosurgery, Wellington Regional Medical Center, Wellington, USA

**Keywords:** spinal subdural hematoma, spontanous spinal subdural hematoma, antiplatelet subdural hematoma

## Abstract

Spontaneous spinal subdural hematomas (SSDHs) are rarely encountered in clinical practice. In this paper, we report a case of a 70-year-old female who presented to the Emergency Department with symptoms of mid-epigastric pain radiating to her mid-scapular region. Her workup demonstrated mildly elevated cardiac troponin I levels and electrocardiogram (ECG) leads V3-V5 ST-depressions. She was subsequently treated through the non-ST elevation myocardial infarction (NSTEMI) protocol and given an antiplatelet agent. The next morning, she developed bilateral lower extremity paresthesias, progressive left lower extremity weakness, and urinary retention. Magnetic resonance imaging (MRI) of the spine demonstrated a hematoma in the thoracic region resulting in spinal cord compression. The patient underwent surgical decompression. After the decompression of the thoracic spinal cord, the patient’s neurological symptoms gradually improved and she regained lower extremity function. This report brings to light a very unusual presentation of an uncommon clinical entity. To the best knowledge of the authors, spontaneous SSDH presenting as myocardial injury and subsequently exacerbated by anticoagulation therapy has not been reported in the literature to date.

## Introduction

First described by Schiller et al. in 1948, under 1000 cases of spinal hematoma have been reported in the medical literature [1]. Those of the spontaneous variety and localized to the subdural space are even more scarce, although they represent a significant source of morbidity [2,3]. Pathogenesis of spontaneous spinal subdural hematomas (SSDHs) is poorly understood. Many hypotheses have been proposed indicating that rupture of the valveless radiculomedullary bridging veins is to blame while others indicate a possibility of a “pseudo-subdural hematoma” with origins in the subarachnoid space [4,5]. In this paper, we report a female who presented with an SSDH and discusses the possible pathophysiology of her illness. Moreover, we call into question the parameters of cardiac troponin elevations required for the justification of a myocardial infarction.

## Case presentation

Patient is a 70-year-old Caucasian female who presented to the ED with a chief complaint of mid-epigastric pain which started the previous morning. She described the pain as sharp and constant, radiating to her mid-scapular region with some suprapubic discomfort. She reported several episodes of non-bilious, non-bloody emesis which were exacerbated with each meal attempt. She denied any headaches, diaphoresis, or dyspnea on admission.

The patient’s past medical history was significant for hepatitis C. She denied any history of hypertension, hyperlipidemia, diabetes mellitus, coronary artery disease, or cerebrovascular accidents. Her surgical history consisted of a hysterectomy, appendectomy, right lower extremity surgery, and laser eye surgery. She admitted to having a course of three epidural injections for lumbago 15 years ago and she denied any prior surgical intervention of the central nervous system. She additionally denied any history of coagulopathies or vascular abnormalities, such as arteriovenous malformations (AVM), in her personal and family history. Her physical exam on admission was unremarkable. She demonstrated no muscle weakness or sensory abnormalities and no focal neurological deficits. She denied any significant trauma history.

The patient’s vital signs on admission were significant for a heart rate of 117 and her first set of lab work was significant for hemoglobin of 16.8, mild leukocytosis at 14.3, hypokalemia at 2.9, hyperglycemia at 146, anion gap of 15.3 and Troponin I of 0.52 (normal 0-0.4 nanogram per milliliter). The remainder of her vital signs, complete blood count, basic metabolic panel, coagulation panel, and acute cardiac enzymes were within normal limits. Pairing these values with an ECG demonstrating ST depressions at Leads V3, 4, and 5, a diagnosis of NSTEMI was made by the admitting physicians. As part of the protocol, the patient received clopidogrel 75 mg.

**Figure 1:**
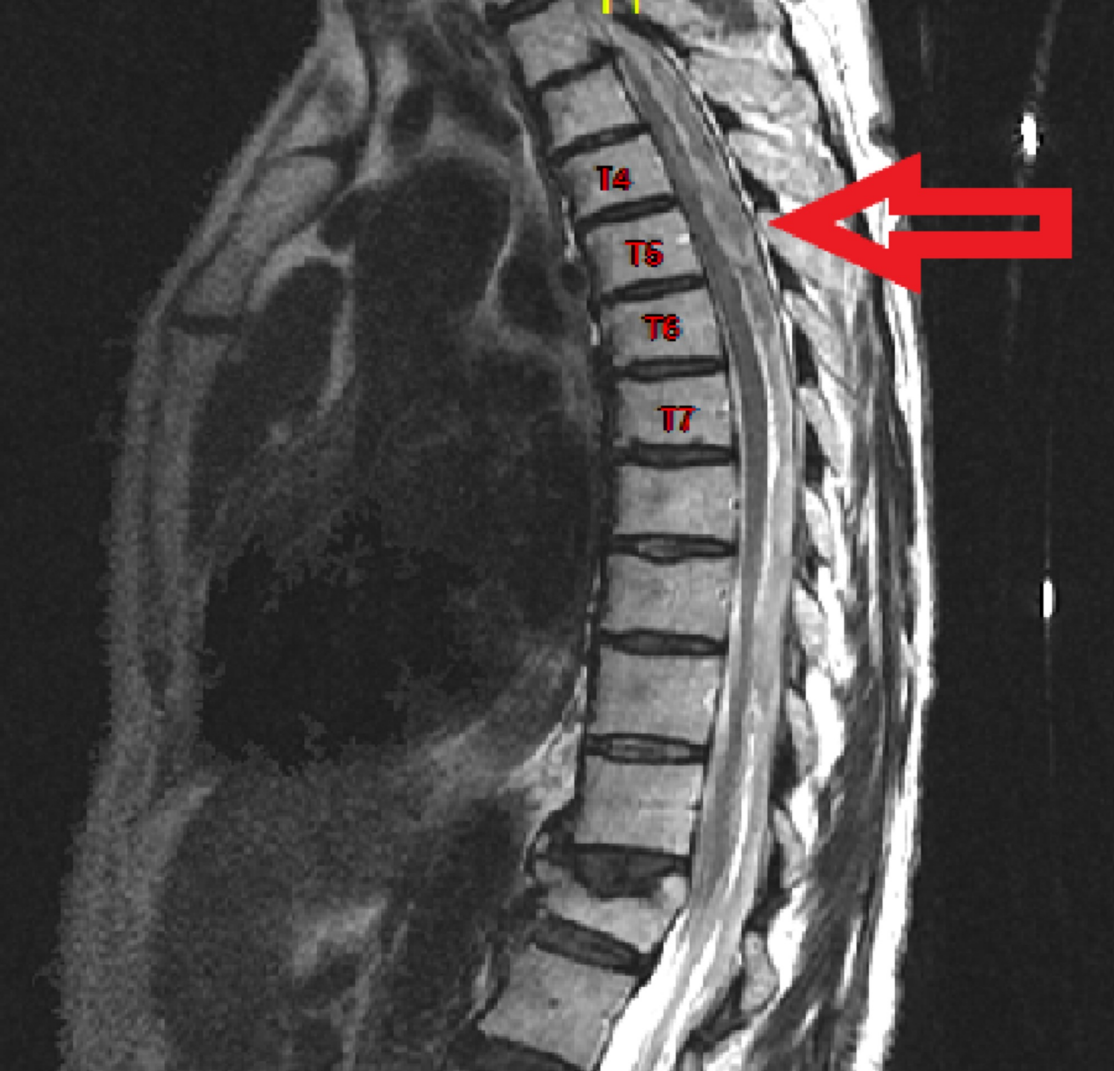
Preoperative T2 sequence MRI, midsagittal cut, of the thoracic spine demonstrating dorsal lesion centered at Thoracic 5 causing spinal cord compression

The following morning, the patient complained of new-onset numbness of the bilateral lower extremities, paresis of the left lower extremity and urinary retention. The neurological exam was significant for muscle strength grade 3/5 in bilateral lower extremities with normal deep tendon reflexes. She was also noted to have diminished sensation below the T6 dermatome to light touch and pinprick; proprioception in the lower extremities was diminished as well. Given these new findings, the medical team consulted Neurosurgery. An MRI of the thoracic spine, as seen in Figure 1, demonstrated a dorsal mass in the spinal canal with significant spinal cord compression at T5/6.

Given the recent clopidogrel usage, a platelet function assay (PFA) was obtained which was consistent with platelet inhibition (196). Platelets were administered after which a repeat assay revealed a normalized value of 131. The patient was taken for urgent surgical decompression and evacuation.

**Figure 2:**
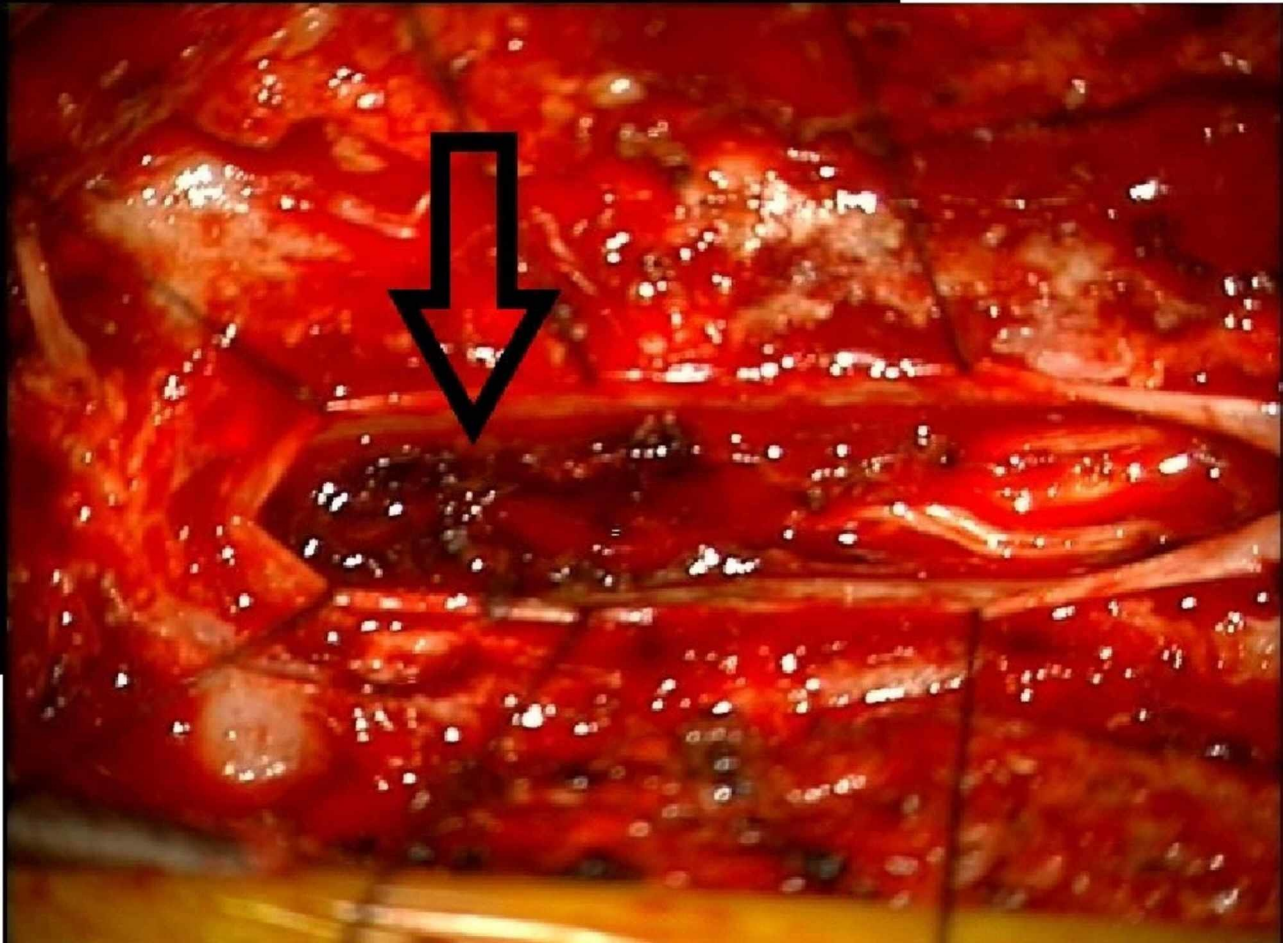
Intraoperative findings of acute on chronic subdural hematoma

After appropriate precautions were taken for the management of an uncontrollable bleed, the incision was made from T3 to T6 since this was the location of significant cord compression. Upon entering the spinal canal, the dura was visualized and the possibility of epidural mass was excluded. The dura was incised and the subdural mass was visualized; a frozen pathological report confirmed it to be a non-malignant hematoma. As visualized in Figure 2, a mixed acute on chronic hematoma was found with some portions very adherent to the spinal cord. Meticulous microdissection was needed for adherent portions and the hematoma was evacuated in a piecemeal fashion. Pulsatility of the spinal cord was visualized once the mass effect was relieved. There was also the identification of an enlarged vein, which was subsequently cauterized with the bipolar forceps. Finally, irrigation with an angiocatheter using normal saline and bacitracin was performed. Xanthochromic fluid was seen and evacuated until a clear return of irrigation was visualized to confirm no acute bleeding. At this time, the evacuation was considered complete. The dura was closed in a watertight fashion and the surgical wound was closed primarily.

On postoperative day 1, the patient exhibited some improvement in lower extremity motor function with grade 4/5 muscle strength. Upon discharge from the hospital, the patient exhibited a grade 4/5 muscle strength on the left and grade 5/5 on the right; however, she was still having urinary retention and required continued urinary catheterization. Furthermore, she had improvement in the chest pain with which she initially presented. The remainder of her inpatient stay was unremarkable, and she was discharged from the hospital to acute inpatient rehabilitation.

The patient had a diagnostic spinal angiogram performed as an outpatient to rule out a possible occult spinal AVM which was found to be negative. Overall, she made a good neurological recovery and now ambulates independently.

## Discussion

The etiology of the spinal subdural hematoma was not identified during the surgery. It is still undetermined whether the hematoma was present on admission or it developed or worsened secondary to antiplatelet therapy. However, given the location of the mass and its impingement on thoracic cord, as well as findings of chronic blood components intraoperatively, it is very likely that a small spinal hematoma was causing radicular pain in the mid-thoracic region which caused the initial epigastric discomfort and subsequent ED presentation; this hematoma may have expanded with antiplatelet therapy to cause sudden neurological deficits. The patient did not have any falls or trauma in the 6-10 hours she was in the hospital with a completely intact neurological exam to the development of paraplegia. However, it is possible that the patient had a remote fall or minor trauma that she failed to recall which may have caused a small SSDH in the outpatient setting. Mohammed et al. described a similar case of a patient who presented with central chest pain, normal ECG, elevated troponins and was treated with dual antiplatelet therapy only to unmask a spontaneous spinal epidural hematoma (SSEH) [6]. Similar to our presented case, that patient had a good recovery after surgical decompression, but to the authors’ best knowledge, this is the first case of a spontaneous SSDH uncovered by NSTEMI treatment. 

The patient’s back pain on admission is consistent with the presentation of a spinal hematoma as described by Michon in 1928 as “le coup de poignard”, or knife stabbing the spine [7]. Although typically presenting as radicular back pain associated with symptoms of myelopathy such as paresthesia, progressive paresis, bowel or bladder dysfunction, SSDH can at times present with vague or unusual symptoms like vaginal or perineal pain, and pure hemiparesis [8-11]. However, de Beer et al. and Domenicucci et al. both found that motor deficit was the most common presentation for SSDH [12,13].

Possible sources of SSDH include, but are not limited to, arteriovenous malformations (AVM), blood dyscrasias, coagulation abnormalities, central nervous system malignancy, and spinal puncture [10,13]. One study found that more than half of SSDH were related to anticoagulation and iatrogenic causes like lumbar puncture or epidural anesthesia [13]. In a review of 151 patients with nontraumatic acute SSDH, 46% had been treated with anticoagulation or had coagulopathies, however, several cases have been reported where the patients had no prior anticoagulation or underlying medical conditions [2]. Furthermore, the evidence in previous medical literature does not support mere anticoagulation as enough to cause a spinal hematoma. Some authors suggest that old age and fragility of the blood vessels is a crucial predisposing factor [14]. Oldenkott et al. claimed that “locus minoris resistentiae” is needed for the initial bleed [15]. Our patient was not on any anticoagulation medication before the event and her coagulation studies were within normal limits on admission.

The etiology of non-traumatic SSDH is still debated in the medical literature. The theory of ruptured subdural veins has met much criticism due to the deficiency of subdural veins in the spine. Rader proposed that a sudden sharp increase in abdominal and intrathoracic pressure led to increased intravascular pressure in the spinal subdural space, creating a differential between extravascular and intravascular compartments and subsequent tearing of spinal vessels [16]. Others have indicated that rupture of extra-arachnoidal vessels that run along the inner surface of the dura could cause SSDH [17]. An alternate hypothesis is an initial subarachnoid hemorrhage (SAH) diffusing through the arachnoid membrane into the subdural space, with a subsequent clearance of the SAH by cerebrospinal fluid, leaving only the SDH [4]. The application of the latter theory to our clinical case is more likely given the visualized adherence of the hematoma to the spinal parenchyma.

It can be very challenging to distinguish a myocardial infarction (MI) secondary to ischemia from increased oxygen demand or decreased supply from troponin release due to non-coronary disease [18]. Discussion with the cardiologist regarding the elevated Troponin I levels yielded the possibility of hypoxia secondary to increased cardiac demand as the most likely source. There is currently a lack of definite parameters for which physicians declare myocardial infarction given a rise in cardiac troponin I levels. MacRae et al. demonstrated that a rise in >20% is practical to declare infarction, while Agewall et al. found that >30% delta change of cardiac troponin I (cTNI) improved specificity and risk assessment [18,19]. It is, however, difficult to distinguish myocardial ischemia secondary to coronary artery disease versus demand ischemia [19].

An MRI was needed to visualize the hematoma in our case. A CT Angiogram of the chest was performed the day prior to rule out the possibility of the pulmonary embolism and no report was made by the radiologist regarding a mass or blood within the spinal canal. A caveat to this conjecture is that the mass had not yet exhibited its neurological deficits so it may have been radiographically invisible, or even physically absent. Discussion with the radiologist indicated that the hematoma was most likely present when reexamining the images in retrospect but would have been impossible to report with certainty during the initial read on CT given the rarity of a spinal hematoma. Given the ease of visualizing CSF versus blood on T2 weighted imaging, MRI is recommended by the authors as the gold standard of visualizing hemorrhage in the spinal canal. MRI findings in SSDH vary depending on oxygenation and age of the clot [20]. Within 24 hours of symptom onset, hematoma is visualized as a space-occupying lesion within the dura, isointense on T1WI and hyperintense on T2WI, but generally, after 24 hours will appear as high signal on T1WI and low signal on T2WI [20].

## Conclusions

The authors would conclude that the hematoma caused the patient’s presenting complaint and the bleeding was further exacerbated with the antiplatelet therapy. The elevated cardiac enzymes and location of pain put myocardial injury very high on the diagnostic differentials, but unfortunately the treatment protocol most likely led to further deterioration. Fortunately, the physicians decided to explore neurological sources, and the hematoma was discovered on imaging before further permanent damage. It is unknown whether the presenting neurological deficits would have resolved on their own with time, but given the sudden rate of deterioration paired with the risk for permanent damage, the authors believe appropriate measures were taken to insure the best outcome for the patient. To date, the prognosis looks promising for the patient.
